# The Relation of Cancer Risk with Nitrate Exposure in Drinking Water in Iran

**Published:** 2019-02

**Authors:** Maryam MORADNIA, Mohsen POURSADEGHIYAN, Amir Hossein MAHVI, Masoud PANAHI FARD

**Affiliations:** 1. Student Research Committee, School of Health, Isfahan University of Medical Sciences, Isfahan, Iran; 2. Health in Emergency and Disaster Research Center, University of Social Welfare and Rehabilitation Sciences, Tehran, Iran; 3. Center for Solid Waste Research, Institute for Environmental Research, Tehran University of Medical Sciences, Tehran, Iran; 4. Department of Environmental Health Engineering, School of Public Health, Ahvaz Jundishapur University of Medical Sciences, Ahvaz, Iran

## Dear Editor-in-Chief

Short-term exposure to nitrate concentration at or higher than the standard level (45 mg/L as NO-3) is serious threat health when conditions result in nitrosation within the human body. Some of the N-nitroso compounds that could be formed in human body organs under these conditions are known carcinogens (1, 2).

In this study, some provinces of Iran with high level of nitrate concentrations in drinking water supplies were selected and excess risk of cancer (ER) related to nitrosamine that formed by nitrate consumed through drinking water was estimated by Monte Carlo analysis and then the results were compared with the rate cancer prevalence. A risk level of cancer provides an estimation of additional cancer incidence in an exposed population that can be expected. A non-threshold model was applied for the estimations of cancer risks, as it represented the worst-case dose-response at low doses. The model supposed that health risk has linear relationship with both the carcinogenicity and the daily dose of a specific nitrosamine (3).

Different guidelines recommend regulation of carcinogens in drinking water at a level which additional cancer risk over a lifetime is essentially negligible. WHO authorities apply a risk level of 1 in 100,000 (4). Moreover, in present study a 10^−5^ risk level was applied to carcinogen risk assessments)

The concentration of nitrate in drinking water supplies of Iran provinces and ER-related to that concentration based on their population exposed are presented in Table 1.

**Table 1:** The concentration of nitrate in drinking water supplies of Iran and the ER related to nitrate

***No.***	***Province’s center***	***Population***	***Nitrate concentration (mg/l)***	***References***	***Excess risk of cancer***	***Position on Iran’s map***
1	Ardabil	1248488	57.62	(5)	1.0×10^−5^	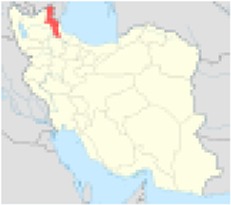
2	Tehran	12183391	87.5	(6)	2.4×10^−5^	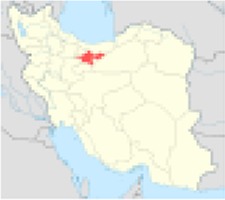
3	Razavi Khorasan	5994402	74.4	(7)	1.7×10^−5^	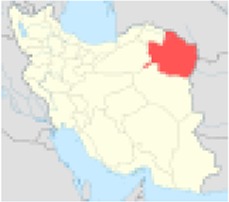
4	Khuzestan	4531720	59	(8)	1.1×10^−5^	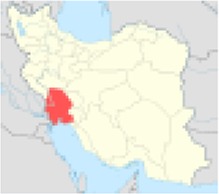
5	Fars	4596658	72	(8)	1.6×10^−5^	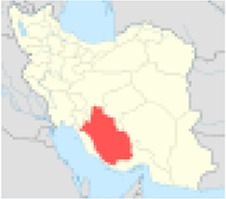

For the maximum protection of human health from the potential carcinogenic effects contributed to exposure of carcinogen N-nitroso compounds, the contaminant concentrations in water should be zero based on the non-threshold assumption for this chemical. However, zero level may not be attainable at the present time. The estimated cancer risks for the province’s center including Tehran, Razavi Khorasan, Fars, Ardabil, and Khuzestan were in the no negligible range set by WHO. Furthermore, the results of many previous studies in Iran made clear the significant association between cancer prevalence rates and nitrate exposure through drinking water. It is found the rate increased of cancer cases in Shiraz (center of Fars Province) from 18% to 81% from 1998 to 2005 (9). A series of reports indicated high incidence of gastric cardia adeno-carcinoma, especially in north, northwestern and southwest provinces of Iran. Ardabil, a northwestern province, had the highest incidence of gastric cancer in Iran. As well as the Tehran metropolitan area also had high rates of cancer (especially gastric cancer).

Furthermore, the prevalence of non-cardia cancer in Khuzestan, southwest of Iran was reported high (8, 10). These reports confirm the results obtained from the present study. According to the risk of 10^−5^ which indicates a probability of one additional case of cancer for every 100,000 people exposed, the probability of additional case of cancer is going up when the number of people exposed increase.

These provinces are more exposed to additional cancer risk related to nitrosamine that formed by nitrate consumed through drinking water. Furthermore, a probability of additional cases of cancer in high population is more. There was a significant association between cancer prevalence and exposing to impermissible level of nitrate in drinking water.
